# Process Evaluation of a Secondary School‐Based Digital Behaviour Change Intervention to Improve Toothbrushing: The BRIGHT Randomised Controlled Trial

**DOI:** 10.1111/cdoe.13019

**Published:** 2024-11-25

**Authors:** Sarab El‐Yousfi, Nicola Innes, Ian Kellar, Caroline Fairhurst, Hannah Ainsworth, Ivor Chestnutt, Peter Day, Donna Dey, Sue Pavitt, Mark Robertson, Katie Whiteside, Zoe Marshman

**Affiliations:** ^1^ School of Clinical Dentistry University of Sheffield Sheffield UK; ^2^ School of Dentistry Cardiff University Cardiff UK; ^3^ Department Psychology University of Sheffield Sheffield UK; ^4^ York Trials Unit, Department of Health Sciences University of York York UK; ^5^ School of Dentistry University of Leeds Leeds UK; ^6^ Community Dental Service Bradford District Care NHS Foundation Trust Bradford UK; ^7^ School of Humanities, Social Sciences and Law University of Dundee Dundee UK; ^8^ School of Dentistry University of Dundee Dundee UK

**Keywords:** behaviour change, dental health, mHealth, oral health, process evaluation, school‐based intervention, text messages

## Abstract

**Objectives:**

The aim was to conduct a process evaluation of a multicomponent behaviour change intervention to reduce dental caries in secondary school children in the UK. The intervention was evaluated in the BRIGHT randomised controlled trial which investigated clinical and cost‐effectiveness. The trial involved 4680 participants from 42 schools with a follow‐up period of 2.5 years. Schools with an above‐average proportion of free school meal (FSM) eligibility, an indicator of low household income, were recruited. The intervention, an oral health classroom‐based session (CBS) delivered by school staff and twice‐daily text messages aimed to improve toothbrushing frequency with fluoride toothpaste.

**Methods:**

Key components of process evaluations were examined: Implementation (fidelity, dose delivered, adaptations and reach), mechanisms of impact (acceptability and dose received) and influential contextual factors. Data collection ran alongside that of the outcome evaluation. Mixed‐methods data collection comprised pupil self‐reported questionnaires, staff feedback questionnaires, CBS and text message delivery logs and semi‐structured interviews/focus groups with school staff and pupils. Quantitative data were summarised descriptively, while framework analysis was applied to the qualitative data.

**Results:**

The intervention was generally implemented as intended, albeit with some schools not confirming CBS delivery and a technical problem resulting in text messages being stopped prematurely. Some adaptations to the CBS were made by school staff. In terms of reach, 21.9% (*n* = 1025) of participants were FSM‐eligible. At baseline, 77.6% (*n* = 3631) of randomised participants reported brushing at least twice daily with no difference over time in the social cognitive determinants of toothbrushing behaviour. The intervention was generally found to be acceptable with varying levels of participant responsiveness reported. The actual dose received was unclear; some schools did not provide a CBS attendance register, and some participants reported blocking or muting the text messages.

**Conclusions:**

This evaluation raises the question of whether the intervention dose and quality of delivery were sufficient to support the required behaviour change mechanisms. Moreover, a high proportion of participants brushed twice daily at baseline; this also calls into question the intervention's ability to bring about significant change. The trial findings did not favour the implementation of the two‐component intervention (CBS and text messages) within a school setting. However, with oral health as part of the general health school curriculum, the BRIGHT CBS could be adopted within the UK curriculum as it was co‐developed with young people and was found acceptable to pupils and teachers.

**Trial Registration:**

ISRCTN number: 12139369

## 1 Background

Oral health promotion efforts in the UK have largely been directed at preschool or primary‐age children, with few initiatives aimed at improving the oral health of adolescents [1]. Moreover, school‐age pupils' interventions have mainly focused on oral health education without being informed by behaviour change theory [2]. The use of mobile health (mHealth) for delivering health interventions has increased [3], and health behaviour change interventions using digital technology have been recommended particularly for young people [4].

The ‘Brushing RemInder 4 Good oral HealTh’ (BRIGHT) trial was a multicentre, school‐based, assessor‐blinded, two‐arm cluster‐randomised controlled trial (RCT) investigating the clinical and cost‐effectiveness of a digital behaviour change intervention for pupils (11–16 years) in UK secondary schools with above national average proportion of children eligible for free school meals (FSM) [5]. FSM eligibility was used as an indicator of low income [6]. The multicomponent, complex intervention was designed to improve the frequency of toothbrushing with fluoride toothpaste and lead to a reduction in dental caries. It consisted of a 50‐min classroom‐based session (CBS), followed by text messages delivered twice daily to participants' mobile phones until the end of the trial or when participants requested the messages to stop. The intervention was informed by behaviour change theory and was co‐designed using a six‐step process involving young people, parents and school staff. The CBS lesson plan and teaching resources were developed to suit the requirements of the curricula and ensure they were appropriate and acceptable to be delivered without additional training. A text message schedule was developed with young people along with a 2‐week pilot that led to offering specific timing options for message delivery and the option to stop and restart the messages.

The BRIGHT trial protocol, intervention development, pilot study, baseline data and main findings have been described elsewhere [5, 7, 8, 9, 10]. In brief, the trial found no statistically or clinically significant difference between the control group (no text messages and standard lessons) and the intervention group in the primary clinical outcome of at least one treated or untreated carious lesion in any permanent tooth at the 2.5‐year follow‐up. There was, however, evidence of a positive effect on twice‐daily toothbrushing at 6 months and a benefit of the intervention among FSM‐eligible pupils in terms of caries prevalence. This paper focuses on the mixed‐methods process evaluation which was undertaken alongside the trial.

## 2 Methods

### 2.1 Process Evaluation

Guided by the UK Medical Research Council (MRC) framework [11], the three key process evaluation components of implementation, mechanisms of impact and context were examined. There is a lack of consistent definitions and considerable overlap for these components [12]. Additionally, there is no consensus on the key subcomponents that constitute implementation [11]. A summary of how the components were operationalised is provided in Table 1.

**TABLE 1:** BRIGHT process evaluation components.

Process evaluation components	Descriptor for BRIGHT intervention
Implementation	Delivery of the intervention in practice
Fidelity	The degree to which the two intervention components (CBS and text messages) were delivered in line with the protocol
Dose delivered	Number of CBS lessons delivered and text messages sent
Reach	The extent to which the target population, according to the eligibility criteria, particularly in terms of free school meal status, participated in the intervention
Adaptations	Any changes made to the CBS for a better fit with the school context. Text messages were not adaptable as automated from a central source
Mechanisms of impact	How participants interact with intervention mechanisms to generate behaviour change
Dose received (exposure)	The extent to which participants were engaged with the intervention: Number of participants who attended and were engaged with the CBS and the number of text messages not only received but also read
Dose received (acceptability)	The degree of acceptability of the intervention components for participants
Context	Contextual factors potentially affecting implementation, intervention mechanisms and intervention outcomes

#### 2.1.1 Implementation

Intervention implementation included the following subcomponents: Fidelity (adherence and quality), dose (what the programme delivered), reach (who participated) and adaptations [11]. For a detailed description of the subcomponents, see Appendix S1.

#### 2.1.2 Mechanisms of Impact

This component explored how the intervention potentially generated behaviour change by how participants responded to and interacted with the intervention [11]. Participant responses are commonly measured in terms of the dose received and acceptability [11] as interventions cannot succeed if unaccepted by participants [13]. Intervention development was informed by the intervention's causal model which included social cognitive toothbrushing determinants such as self‐efficacy, attitude (social norms, outcome expectancy and risk perception), intention, coping planning and action planning. Behaviour change techniques and intervention strategies were developed to target each of these determinants (Appendix S2).

As described in the Implementation section, dose received or ‘exposure’ refers to the extent to which participants actively engage with and are receptive to the materials or resources [14]. This would have required measuring the number of text messages received and read by participants. For the CBS, this meant assessing participants' active engagement with the activities.

This process evaluation explored how participants interacted with the intervention by assessing acceptability, dose received and social cognitive toothbrushing determinants.

#### 2.1.3 Context

The intervention context was explored to identify any potential interactions with intervention mechanisms and implementation and how it may have influenced and interacted with the delivery and functioning of the intervention and its outcomes. This involved examining the broader school culture and included external factors such as the wider context, school structure, curriculum and possible contamination within the school.

### 2.2 Recruitment and Consent

The process evaluation included a quantitative component and a qualitative component. The quantitative component involved the sample for the outcome evaluation. The qualitative component involved a smaller, purposive sample of school staff and pupil participants.

The sample for the quantitative component was recruited from the 42 participating schools and included pupils aged 11–14 years from years 7 and 8 (England and Wales) and S1 and S2 (Scotland). Written consent was obtained from all participants.

Participants who completed the baseline questionnaire and dental assessment received a £10 gratitude voucher, and those who completed the final follow‐up questionnaire and dental assessment received another £5 voucher.

The qualitative sample of pupil participants was drawn from six schools across the trial sites employing purposive maximum variation sampling using the variables of year group, sex, age and regional location. Pupils were invited to take part in focus groups and returned a reply slip to school to express their interest. There were no dropouts from those who registered interest. Staff members involved in CBS delivery were invited and offered the option of a face‐to‐face or telephone interview. A total of 14 staff members expressed interest, but two interviews did not take place due to difficulties with scheduling. Written consent was obtained for focus groups and face‐to‐face interviews and verbal consent for telephone interviews. Interview participants (pupils and staff) received a £10 gratitude voucher.

### 2.3 Data Collection

Data collection ran alongside the outcome evaluation. Quantitative data collection occurred in both the control and intervention arms of the trial; qualitative data collection occured only in the intervention arm.

#### 2.3.1 Quantitative

The delivery of the two intervention components was monitored and documented, and data were collected from schools and the TextApp software.

##### 2.3.1.1 School Records and Publicly Available Sources

Schools provided sociodemographic data for pupil participants such as sex, year group and current eligibility for FSM. School‐level data were also captured from publicly available sources, such as government reports on the proportion of children eligible for FSM.

##### 2.3.1.2 CBS Delivery Information Provided by Schools

Schools completed intervention delivery logs confirming CBS delivery with the delivery date and the person who delivered it (name and professional capacity). A register of pupil attendance was also provided.

##### 2.3.1.3 Text Message Records

The number of text messages sent was recorded using TextApp software. Participants who no longer wanted to receive text messages could text back ‘STOP’ (free text); this allowed recording the number of participants who withdrew and when. They could text back ‘START’ if they changed their mind. The records collected the dosage delivered and other metrics (Box 1). However, there was no facility to confirm if messages were received and read, so collecting the dosage received was not feasible. Replies received were also monitored for safeguarding purposes and managed in line with trial‐safeguarding protocols.

BOX 1 **Text message metrics.**

**Table:** 

Number of text messages sent per participant Number of text messages undelivered per participant The number of participants texting back STOP and START and when this occurred Total number of replies to text messages The number of replies per participant/per message sent Timings between message delivered and reply Number of participants who reported a change of telephone number

##### 2.3.1.4 Pupil Questionnaires

Participants completed questionnaires at several time points: Baseline, 6 months, 1 year and 2.5 years post‐CBS delivery. The questionnaires assessed the frequency of toothbrushing and toothbrushing determinants (motivational and volitional factors). These determinants were included based on the intervention's causal model and included self‐efficacy, attitude (social norms, outcome expectancy and risk perception), intention, coping planning and action planning. The questions (Appendix S3) were adapted from those used and validated in earlier studies regarding other oral health behaviours [15, 16].

#### 2.3.2 Qualitative

##### 2.3.2.1 Interviews

Interview topic guides were developed (Z.M. and S.E.) and informed by the process evaluation framework [11] and the theoretical framework of acceptability [13]. Interviews were facilitated by experienced qualitative researchers from different disciplines (dentistry and social science) and different genders (Z.M.[F], S.E.[F], H.L.[F], M.R.[M] and R.H.[F]). Four of the focus group interviews were facilitated by three peer mentors. All interviews were audio‐recorded and transcribed verbatim. Interviews took place between June 2019 and November 2019. Focus group interviews took place within 4–6 months post‐CBS delivery.

##### 2.3.2.2 CBS Staff Feedback Questionnaires

Staff members involved in CBS delivery completed a lesson feedback questionnaire (*n* = 14) which included open‐ended responses.

A summary of the process evaluation components assessed and their respective data sources is presented in Table 2.

**TABLE 2:** Process evaluation components and data sources.

Research objective	Process evaluation measure	Data source	
Implementation	Fidelity Dose delivered Adaptations Reach	Fidelity	CBS CBS delivery information provided by schools CBS staff feedback questionnaires Interviews and focus groups Text messages Text message records (TextApp software)
		Dose delivered	CBS CBS delivery information provided by schools CBS staff feedback questionnaire Interviews and focus groups Text messages Trial text message records (TextApp software)
		Adaptations	CBS staff feedback questionnaire Interviews and focus groups
		Reach	Trial records Publicly available resources School records
Mechanism of impact	Dose received Acceptability Mediators causal model	Dose received	CBS CBS pupil attendance information provided by the schools CBS staff feedback questionnaire Interviews and focus groups Text messages Interviews and focus groups
		Acceptability	Interviews and focus groups
		Mediators	Self‐report pupil questionnaires Interviews and focus groups
Context			Interviews and focus groups Publicly available sources Trial records

### 2.4 Data Analysis

Process evaluation data were analysed while blind to the trial outcome data [11]. Qualitative and quantitative data were analysed separately and iteratively. The two data sets were then synthesised and interpreted together to provide a more nuanced understanding of the main outcome findings of the trial.

#### 2.4.1 Quantitative

Quantitative data were summarised descriptively and presented as continuous measures (means, standard deviations (SDs)) and categorical data (counts and percentages). The pupil questionnaire items assessing motivational and volitional factors had a four‐point response scale (1 = Not true at all, 2 = Not true, 3 = True and 4 = Definitely true), except for the intention item, which had six responses ranging from ‘Never’ to ‘More than three times a day’. For motivational and volitional factors with more than one item (attitude and action planning), a scale was produced using the response mean.

A complier average causal effect (CACE) analysis was conducted as part of the main clinical effectiveness analysis for the primary clinical outcome of the presence of at least one treated or untreated carious lesion in any permanent tooth. It assessed the impact of compliance with the intervention defined at the participant level in three different ways: attending the CBS, attending the CBS along with receiving at least 50% of text messages a week for the first 12 weeks and the number of text messages sent. Subgroup analyses also examined FSM status and its relation to intervention outcome.

#### 2.4.2 Qualitative

All interview data (individual and focus group) were analysed using Framework Analysis [17]; this involved (1) familiarisation, (2) identifying initial themes, (3) labelling the data, (4) sorting the data by theme and (5) mapping and interpretation. An initial framework was developed adopting inductive and deductive approaches to explore the a priori themes identified from the literature and new themes derived from the data. Field notes were used to help interpret the data. The analysis of the acceptability data was undertaken primarily by two experienced doctoral researchers and refined with discussion with a senior experienced qualitative researcher/academic. Nvivo12 QSR was used for data management and retrieval of raw data to support analysis and write‐up. Further refinement of themes was undertaken by one of the postdoctoral researchers, and any discrepancies were discussed and resolved. This process strengthened inter‐rater reliability and credibility ensuring trustworthiness. Recruitment continued until data saturation was reached, which was defined as the stage at which no new information from participants added to the overall interpretation of the evaluation.

## 3 Results

The evaluation was implemented in 42 schools with a total of 4680 randomised participants (intervention, *n* = 2262; control, *n* = 2418). A baseline pupil questionnaire was at least partially completed for 4626 randomised participants (98.8%: intervention *n* = 2234, 98.8%; control *n* = 2391, 98.9%).

Focus group interviews (*n* = 6) were conducted with 50 intervention arm participants (25 girls and 25 boys) aged 11–13 years from six schools. The focus groups were held in school and lasted, on average, 45 min. Semi‐structured interviews (*n* = 12) were undertaken with school staff: Teachers (*n* = 6), learning managers (*n* = 2) and those in senior leadership roles (*n* = 4). Interviews were held at school (*n* = 4) or via telephone (*n* = 8) and lasted, on average, 20 min.

### 3.1 Intervention Implementation (Fidelity, Dose Delivered, Reach and Adaptations)

The intervention was generally implemented as intended. Some schools, however, did not confirm CBS delivery, and some technical challenges resulted in all texts being stopped prematurely. In terms of reach, 21.9% (*n* = 1025) of participants were eligible for FSM.

The CBS was delivered in 39 of the 42 participating schools. One school reported the CBS was not delivered, and two schools did not respond; therefore, the CBS was considered as undelivered. It was reportedly delivered as a single 50‐min session in 1 day as intended, except in one school where the CBS was delivered over three sessions throughout the week for timetabling reasons.

Of the 39 schools, 30 provided a CBS register of attendance. For the school where the CBS was delivered over three sessions, only participants who attended all three sessions were counted as having attended the CBS. For the remaining nine schools, the assumption was made that all intervention participants attended the session; therefore, the estimated total may be a slight overestimation. An estimated 89.1% (*n* = 2016) of intervention participants attended the CBS. One school only delivered it to two out of the eight classes in the intervention year group and did not provide a CBS register of attendance.

There was some partial contamination in the control group as one school mistakenly began to deliver the CBS to the wrong allocated year group due to miscommunication at the school. This occurred in the school that delivered the CBS over three sessions. We could not establish how much of the CBS was delivered to the control group nor to whom, as no measures for recording attendance were in place for the control group. Therefore, a conservative assumption was made that all control participants (*n* = 69) in this school received the full CBS.

In some cases, the CBS was adapted for the specific context including changes to the delivery method and content. The BRIGHT CBS was designed to be delivered within the classroom; however, some staff members described delivering it as part of a whole‐year assembly for logistic reasons, particularly if they did not have a dedicated PSHE education lesson.

I did it with the whole year group in an assembly…we did it in two sessions. But ours was purely logistical issues to do with the school timetable. … (School staff: 78:1)

Additionally, the lesson plan was adapted to the needs of their pupil cohort (learning style and level) and the duration of the lesson.

That video was a bit sort of immature for the age group, so they found their own videos. (School staff: 39:1)

…we tried to find a way of making it more interactive and a bit more so they could participate a little bit more…. he adapted it and he made a PowerPoint presentation. (School staff: 38:1)

Now that was a stretch to keep that going for 50 min… there wasn't enough content…because classes now are quite fast moving. What we did, it led us onto a good question and answer session where the students were asking and answering each other's questions. (School staff: 39:2)

Furthermore, adaptations were made to gain a sense of ownership and better suit individual teaching style.

…he spent a lot of time looking at that and changing it and making it his own and having some ownership of that lesson to make it run smoothly. (School staff: 36:1)

Text messages were sent to 99.8% (*n* = 2258) of the 2262 participants in the intervention year groups. Texts commenced a median of 5 days (range; 19 to 168) post‐CBS delivery. Messages were sent between 0 and 127 weeks (mean 53.4  weeks, SD 35.4), with between 1 and 1708 texts (mean 694.5, SD 468.9) being sent and just over 70% of sent messages recorded as being successfully delivered. A total of 42.5% (*n* = 962) of intervention participants withdrew by texting back STOP a median of 2.8 months after they began (range 1 day to 30 months). There were 15 safeguarding concerns raised throughout the trial; 87% (*n* = 13) were due to the nature of the content of the text message replies.

Text messages were intended to continue being sent until the 2.5‐year follow‐up post‐CBS delivery or until participant withdrawal. However, due to a technical error with the text provider, messages stopped prematurely on 12 July 2020. At this point, 60.5% (*n* = 1368) of participants had not withdrawn (texted STOP), and most participants had received text messages for more than 10 months. As this error was only discovered 5 months later, the decision was made not to restart the messages.

A total of 48.3% (*n* = 1093) of intervention participants met the CACE analysis criteria of CBS attendance and were receiving at least 50% of the text messages per week for the first 12 weeks. The CACE estimate of the intervention effect was similar to the intention‐to‐treat estimate. The CACE estimate related to the number of texts sent indicated that for every additional text message sent, there was no evidence of a decrease in the likelihood of having a carious lesion. The subgroup analyses indicated a benefit of the intervention among FSM‐eligible participants but not among those who were not.

### 3.2 Mechanisms of Impact (Acceptability, Dose Received and Participant Responsiveness)

Quantitative findings from the pupil questionnaires found no difference over time in the social cognitive determinants of toothbrushing behaviour (Appendix S4).

Intervention acceptability has been described elsewhere [18]. In brief, both intervention components were found overall to be acceptable. Participants reported the lesson was informative, and the text messages were helpful reminders reinforcing the need for twice‐daily toothbrushing. However, some described that the text messages became annoying due to their frequency and repetitiveness and consequently muted or blocked them. This meant the number of text messages sent (dose delivered) did not represent the number of text messages read (dose received).

Participants were informed that the text messages were not meant to be replied to. Despite this, 8461 text responses were received from 61.5% of participants (*n* = 1388), with between 1 (*n* = 360) and 585 (*n* = 1) responses received per participant (mean 6.1, SD 18.4, median 3, mode 1). Excluding the STOP and START messages, there were 7124 free‐text responses received. Most responses (66%) were positive or confirmed their intention to brush their teeth, providing some evidence of engagement with the messages.

Baseline data indicated just over three‐quarters of participants reported brushing twice daily. Indeed, some participants reported that this was already part of their normal routine.

Usually, I remember just to brush my teeth anyway, because it's just habit, but it's been helpful sometimes when I forget to brush my teeth if I'm in a bit of a rush and then I just do it quickly if I get a text. (Pupil: 62:Y8:PS6)

Nevertheless, several participants referred to the usefulness of the intervention in ‘providing general encouragement’ (Appendix S1).

They helped me remember in the night because I didn't use to do it in the night but I do now. (Pupil: 75:Y8:PS6)

Like keeping my teeth like healthier because like I went back to the dentist the other day and they said that it's like my teeth looked really good like better than what they used to look like. (Pupil: 16:S1:PS3)

They also spoke of the value of the CBS in ‘modelling behaviour’ and ‘providing information on consequences’

But like when I watched the video in that, you just like imagine it, “Oh yeah, you do need to brush your teeth properly.” … Like just knowing that if you didn't brush your teeth before that, like all the things that would be building up too…and it would make you like want to brush your teeth. (Pupil: 57:Y7:PS6)

Additionally, participants spoke of the CBS helping them to ‘identify facilitators’ for toothbrushing

Well, like at night time usually like I'll brush my teeth now before I go to like watch something or play something. I'll brush my teeth before then, so then I don't forget about it. And now I want to do it. And that makes it easier. (Pupil: 16:S1:PS3)

While others described how it ‘prompted intention formation’

Because usually like in the night or something like I'll brush my teeth or something. Well, I used to. But then like I started brushing them properly in the night after we got the assembly. (Pupil: 57:Y7:PS4)

Some adaptations such as delivering the lesson as an assembly rather than an in‐class session appear to have curtailed pupil engagement and thus the quality of delivery.

…. especially them being such a big group, the students will have treated it as an assembly rather than a lesson so …an assembly they have to sit in silence and listen. (School staff: 75:2)

And I didn't feel like I could ask questions because there was so many people. (Pupil: 75:Y8:PS7)

Staff and pupils reported varying levels of engagement with the CBS and with different activities of the lesson.

…they were engaged with that task with… the story there. So, I think the lesson all in all, it worked fairly well in terms of pupils. (School staff: 78:1)

When they had to sort of brainstorm about things about tooth decay, …you know, why is brushing your teeth good, what happens when, you know, brush your teeth. They liked the bit where they were engaging, they're thinking, they're working in groups …that was really good. (School staff: 33:1)

At the end it got very boring…because it'd been like really long. (Pupil: 57:Y7:PS6)

Indeed, some pupils could not remember some of the CBS content and activities.

I can't even remember much. But I remember, like they've showed us plaque and cavities and how to stop them from growing on your teeth. (Pupil: 62:Y8:PS3)

I must of found it boring because I don't remember a thing…I can only remember the part about watching TV and forgetting {to brush my teeth}. (Pupil: 16:S1:PS7)

Having the CBS delivered by a staff member whom pupils had built rapport with appears to have facilitated engagement.

Yeah, they did engage. And it was a nice class. I've got to admit. It was a class of kids who I've worked with a lot, and they were very good, so that did help I want to admit. Constantly. It was all the way through, loads and loads of questions (School staff: 36:2)

### 3.3 Context

Baseline data for the randomised participants are presented to provide some context of the intervention in Table 3.

**TABLE 3:** Baseline data summary of sociodemographic characteristics of randomised participants and data from self‐reported questionnaires.

Baseline sociodemographic characteristics of randomised participants (*n* = 4680)	
Age at recruitment, mean (SD)	12.7 years (0.6)
Sex	54.2% female (*n* = 2538)
Eligible for FSM	21.9% (*n* = 1025)
IMD decile of deprivation, mean	England 3.1 (2.4) Scotland 4.4 (2.9) Wales 3.3 (2.2)

Baseline participant data from self‐report questionnaires (*n* = 4626)	
Proportion of participants who brushed their teeth at least twice a day	77.6% (*n* = 3631)

Qualitative findings indicate that school contextual factors influenced intervention delivery. As previously mentioned, adaptations were made by some staff members to better fit the school context.

Wider contextual factors such as government policy also appear to have affected intervention implementation. Schools commonly have competing priorities, and introducing additional educational content not geared toward national qualifications can be challenging. In Scotland and Wales, general health is part of the curricula; however, there is less emphasis on oral health. Current policy in England mandates that oral health is included in the curriculum [19].

However, this policy only came into effect after intervention delivery nonetheless many staff members knew of the imminent inclusion of oral health in the curriculum. Consequently, incorporating the CBS into the PSHE education curriculum appears to have positively influenced CBS acceptability for school staff in England.

…because with the curriculum guidelines, you know, about delivering PSHE…we can build that into the PSHE frameworks to deliver it…now it's part of something they have to deliver anyway, so they might as well use what's provided to them and put that in their lesson plans…we thought we'd put that in place from this year…We completely agree with it…and feel that is a level that's missing, you know, to support students. (School staff: 38:1)

The wider context also included that the trial follow‐up was conducted during the COVID‐19 pandemic. The intervention was designed to establish toothbrushing within daily routines and was linked to specific activities at certain times of the day such as waking up for school. The pandemic brought about changes for most participants related to schooling (online learning), instability of life and lack of routine. The impact of the pandemic could not be quantified.

## 4 Discussion

Overall, the intervention components were delivered as intended. Notably, however, some schools did not confirm CBS delivery, and some adaptations were made to the CBS content and delivery method. The reach of the intervention was found to be good; schools with higher than the national average proportion of FSM‐eligible pupils participated, with 21.9% of participants eligible for FSM. There was some contamination with one school delivering the CBS to some participants in the control year group. Also, due to technical challenges, text messages were stopped prematurely. Furthermore, 42.5% of participants requested to no longer receive text messages at a median of 2.8 months after they started. Messages were sent to participants for an estimated median of 14 months, with just over 70% of messages recorded as delivered. Qualitative findings indicated that despite participants finding the text messages acceptable, some reported blocking or muting them, suggesting they may have experienced boredom and alert fatigue. This is in line with previous studies of mHealth interventions that similarly reported boredom, annoyance, habituation (ignoring messages) and alert fatigue as challenging aspects for long‐term engagement [20, 21, 22]. The actual dose received for the intervention is unclear due to some challenges; it was unfeasible to document how many messages were blocked or muted or for how long. Additionally, some schools did not provide a CBS attendance register, and an assumption was made that all intervention participants attended. An estimated 90% of intervention participants attended the CBS, but this is likely an overestimation.

Importantly, some students were unable to remember certain CBS activities suggesting that they did not fully engage with the lesson. Implementation quality is often a challenging aspect to evaluate [12]. This process evaluation raises the question of whether the intervention dose and the quality of delivery were sufficient to trigger the anticipated behaviour change mechanisms required for behaviour change.

Another important consideration is the intervention context. Baseline demographics show that just over three‐quarters of participants reported brushing twice daily. This also brings into question the ability of the intervention to achieve significant clinical improvements in this sample, since the intended behaviour change would need to occur in the quarter of participants who were not already brushing twice daily. Moreover, follow‐up for the BRIGHT trial was largely conducted during the COVID‐19 pandemic. Besides adversely affecting data collection and follow‐up rates, the pandemic would have undoubtedly brought about changes to the daily lives of secondary school pupils such as remote schooling and having an established routine. This is significant as the behaviour change intervention was designed considering the ‘normal’ daily routines of pupils, i.e., before the pandemic; therefore, this potentially may have hindered the intervention behaviour change mechanisms.

### 4.1 Strengths and Limitations

Several limitations of this study are acknowledged. Intervention participants would most likely have been aware of the recommendation to brush twice daily as reinforced in the CBS, and thus there was a risk of reporting bias to respond more positively in the questionnaires and the focus groups facilitated by study researchers. However, four focus groups were peer‐to‐peer‐facilitated which may have mitigated the risk of reporting bias. There was also the potential for response bias from some participants due to the gratitude vouchers provided for completing questionnaires and participating in the focus group. Moreover, the focus groups were conducted 4 to 6 months post‐CBS delivery, and this may have impacted the ability of members to recall details of the lesson. Also, accurately establishing contamination was challenging as this may have been under‐reported by schools and participants, and not all potential avenues were captured. Another critical limitation acknowledged is the difficulty in accurately reporting the dose received of both intervention components, particularly the text messages. Moreover, implementation quality has a significant impact on study outcomes [23]; however, the quality of the delivery of the CBS was not evaluated. Observation data on CBS delivery may have provided insight into how well it was delivered, but this was not feasible.

To the best of our knowledge, this is the first dental trial to use this detailed methodology. By combining mixed‐methods data on reach, dose and fidelity, the process data provided several important findings. This allowed for a better interpretation of the outcome evaluation and offered some insight and possible explanations for the insufficiency of the intervention, at the 2.5‐year follow‐up, to achieve the intended clinical outcome of lower caries prevalence through significant improvements in toothbrushing frequency.

Notably, at the 2.5‐year follow‐up, there was evidence of a difference in gingivitis with a lower bleeding score in the intervention group. There was also evidence of positive behaviour change on twice‐daily toothbrushing self‐reported at 6 months [9]. Previous behaviour change studies implementing a text message intervention have found improvements in toothbrushing, plaque and bleeding gingivae; however, almost all studies had follow‐up times of 6 months or less. Moreover, most were implemented in clinical settings, for example, with patients undergoing orthodontic treatment, rather than in school‐based settings.

### 4.2 Implications

The findings of this process evaluation are likely to have implications for the development and evaluation of oral health promotion interventions for use in secondary school settings, such as the need for more than one lesson to be given over the pupil's time at secondary school. As well as the development and evaluation of mHealth interventions, particularly text message interventions aimed at young people.

### 4.3 Recommendations

The findings of this trial do not favour the implementation of the two‐component intervention (CBS and text messages) in a school setting. Some challenges with delivering text messages on this scale could potentially be amplified if delivered at scale such as the technical difficulties and staff‐ and cost‐resources required for safeguarding children.

With the incorporation of oral health within the school curriculum across the UK, the tested CBS is available for use and allows schools to meet this learning objective. Further research is recommended to develop approaches to evaluate text message interventions including duration, frequency of messages, interactivity and dose. Also, it would be beneficial to further explore the impact of the CBS in secondary schools and how it can be adapted for young people with additional support needs. Further research is also recommended to explore how individual elements interact in multicomponent interventions.

## Author Contributions

Z.M. and N.I. are the co‐PIs in the BRIGHT trial, designed the trial and wrote the original grant application. Z.M., N.I., I.K. and D.D. were involved in intervention development. H.A. was responsible for day‐to‐day trial management. C.F. wrote the statistical analysis plan and conducted the statistical analysis. P.D., S.P. and I.C. contributed to the trial design and data collection. K.W. was involved in trial coordination and data collection. M.R. contributed to data collection.

Z.M., N.I. and S.E. contributed to the process evaluation design. S.E. and Z.M. contributed to data collection and analysis and wrote the initial draft and subsequent revisions. N.I. and C.F. provided a critical review and editing of the initial manuscript draft. All authors critically revised the manuscript and approved the final manuscript.

## Ethics Statement

The East of Scotland Research Ethics Committee provided ethical approval for the BRIGHT trial (REC reference: 17/ES/0096).

## Consent

Informed consent was obtained from all participants. We confirm that all methods were carried out per relevant guidelines and regulations.

## Conflicts of Interest

The authors declare no conflicts of interest.

## Supporting information


Appendix S1.

Appendix S2.

Appendix S3.

Appendix S4.

## Data Availability

The data set used and/or analysed during the current study is available from the corresponding author upon reasonable request. The full report for the trial can be found at the link on the NIHR HTA website. https://www.fundingawards.nihr.ac.uk/award/15/166/08.
