# Supplementary material for: Process Evaluation of a Secondary School‐Based Digital Behaviour Change Intervention to Improve Toothbrushing: The BRIGHT Randomised Controlled Trial
Source: Community Dent Oral Epidemiol. 2024 Nov 25;53(2):180–9. doi: 10.1111/cdoe.13019 (PMC11892544; doi:10.1111/cdoe.13019)
Supplement: Supplementary file 1 — Appendix S1. [file CDOE-53-180-s003.docx]

#####

##### Appendix 1. Description of the intervention implementation subcomponents.

##### Fidelity

Measures of fidelity include establishing if the intervention was delivered as planned^1^ (adherence) as well as if it was delivered effectively in both ‘the manner and the spirit’^2^ or quality to achieve the intended outcome.^3^ Quality includes features related to the individual delivering the intervention such as competence, skills, enthusiasm, and preparedness for delivery.^4^

Assessing CBS fidelity included monitoring aspects such as the date of delivery, duration of the lesson and whether it was delivered in one session or split across more than one session. For text messages, this involved assessing whether twice-daily text messages were delivered to participants.

##### Dose

Dose delivered and dose received are two different process evaluation components but conceptually similar.^2,5^ Dose delivered refers to the amount or number of intended units of each intervention component that is delivered to participants. It is therefore directly related to intervention implementation and is often determined by the behaviours of those delivering the intervention. Dose delivered included recording the number of texts delivered and for how long, and if the CBS was delivered or not.

Dose received on the other hand, refers to the extent to which participants actively engage with, are receptive to, and/or use materials or recommended resources, also referred to as ‘exposure’.^6^ Measures of the dose received include the proportion of the intervention components received as well as what proportion of the intervention components participants engaged with. Dose received is often considered a subcomponent of the mechanisms of impact.

##### Reach

Reach refers to the extent to which the intervention reached the target population^2^ and is usually measured as the proportion of the target population that attends or participates in the intervention. The BRIGHT trial was aimed at young people, particularly those living in areas of deprivation. The reach of the intervention was therefore assessed by the number of pupils who participated particularly those eligible for FSM.

##### Adaptations

This refers to any changes made to elements of the intervention components for a better contextual fit to enable or improve delivery.

1. Carroll C, Patterson M, Wood S, Booth A, Rick J, Balain S. A conceptual framework for implementation fidelity. *Implementation Science*. 2007;2(1):40. doi:10.1186/1748-5908-2-40

2. Linnan L, Steckler A. Process evaluation for public health interventions and research: an overview. In: *Steckler A, Linnan L, Eds. Process Evaluation for Public Health Interventions and Research*. Vol 28. Jossey-Bass San Francisco; 2002:1-23.

3. O’Donnell CL. Defining, conceptualizing, and measuring fidelity of implementation and its relationship to outcomes in K–12 curriculum intervention research. *Review of educational research*. 2008;78(1):33-84.

4. Lendrum A, Humphrey N, Greenberg M. Implementing for success in school-based mental health promotion: The role of quality in resolving the tension between fidelity and adaptation. In: *Mental Health and Wellbeing through Schools*. Routledge; 2016:53-63.

5. Pirie PL, Stone EJ, Assaf AR, Flora JA, Maschewsky-Schneider U. Program evaluation strategies for community-based health promotion programs: perspectives from the cardiovascular disease community research and demonstration studies. *Health Education Research*. 1994;9(1):23-36.

6. Baranowski T, Stables G. Process evaluations of the 5-a-day projects. *Health Education & Behavior*. 2000;27(2):157-166.
