# Supplementary material for: Process Evaluation of a Secondary School‐Based Digital Behaviour Change Intervention to Improve Toothbrushing: The BRIGHT Randomised Controlled Trial
Source: Community Dent Oral Epidemiol. 2024 Nov 25;53(2):180–9. doi: 10.1111/cdoe.13019 (PMC11892544; doi:10.1111/cdoe.13019)
Supplement: Supplementary file 2 — Appendix S2. [file CDOE-53-180-s004.docx]

Appendix 2. BRIGHT behaviour change techniques and intervention strategies

|  | | |  |
| --- | --- | --- | --- |
| Technique | Definition | Intervention strategies |  |
|  | | |  |
| 1.Provides information on consequences | Information about the benefits and costs of action or inaction, focusing on what will happen if the young person brushes effectively. | Changing attitudes and intention formation:  CBS explaining effectiveness of fluoride toothpaste at improving oral health based on concerns of schoolchildren i.e. appearance, social reasons, health reasons including reducing tooth decay. This information will be reinforced with the SMS |  |
|  | | |  |
| 2.Prompts intention formation | Encouraging the young person to decide to improve their toothbrushing. | Developing intention: CBS encourages development of a personalised brushing plan in the CBS |  |
|  | | |  |
| 3. Prompts barrier identification | Identifying barriers to toothbrushing and plan ways of overcoming them. | Action and coping planning:  Identify barriers and  formulation of coping plans in the CBS |  |
|  | | |  |
| 4. Prompts facilitator identification | Identifying facilitators to toothbrushing and plan ways to use them to overcome barriers. | Action and coping planning:  Action planning in CBS |  |
|  | | |  |
| 5. Provides general encouragement | Supporting the young person to improve their toothbrushing | Intention formation:  Thru CBS  Encouragement through SMS |  |
|  | | |  |
|  | | |  |
| 6. Provides instruction | Telling young people how to brush effectively | Improving self efficacy and action planning:  Video clip and factsheet given during CBS and re-enforced through SMS |  |
|  | | |  |
| 7. Models or demonstrates the behaviour | An expert shows the young person person how to correctly perform toothbrushing. | Improving self- efficacy through video clip and factsheet given during CBS and re-enforced through SMS |  |
|  | | |  |
| 8. Prompts specific goal setting | Involves detailed planning of what the person will do, including a definition of the behaviour specifying twice daily toothbrushing for 2 minutes in terms of where, when and how. | Intention formation and action planning in the CBS |  |
|  | | |  |
| 9. Teaches to use prompts  or cues | Teaching young person to identify cues that can be used to remind them to perform  a behaviour associated with times of day and transitionary spaces | Action and coping planning in the CBS  Reminders of the cues through SMS |  |
|  | | |  |
