# Supplementary material for: Process Evaluation of a Secondary School‐Based Digital Behaviour Change Intervention to Improve Toothbrushing: The BRIGHT Randomised Controlled Trial
Source: Community Dent Oral Epidemiol. 2024 Nov 25;53(2):180–9. doi: 10.1111/cdoe.13019 (PMC11892544; doi:10.1111/cdoe.13019)
Supplement: Supplementary file 3 — Appendix S3. [file CDOE-53-180-s001.docx]

Appendix 3. **Items from pupil questionnaires used to assess motivational and volitional factors related to toothbrushing**

| **Motivational and volitional factors influencing toothbrushing** | **Items from pupil questionnaire** |
| --- | --- |
| Self-efficacy | - I know how to brush my teeth properly |
| Intention | - How often do you want to brush your teeth? |
| Action planning | - I know where and when I will brush my teeth in the morning - I know where and when I will brush my teeth in the evening |
| Coping planning | - I have a plan of how I will make myself brush when I find myself not brushing properly |
| Attitude | - If I brush my teeth twice every day then my teeth will look clean when talking to friends - If I brush my teeth twice every day then my teeth will be healthy - If I brush my teeth twice every day then my teeth will feel good - If I don’t brush my teeth twice every day, I risk getting tooth decay - If I don’t brush my teeth twice every day, I risk my teeth looking dirty - If I don’t brush my teeth twice every day, I might have bad breath |
