# Supplementary material for: Process Evaluation of a Secondary School‐Based Digital Behaviour Change Intervention to Improve Toothbrushing: The BRIGHT Randomised Controlled Trial
Source: Community Dent Oral Epidemiol. 2024 Nov 25;53(2):180–9. doi: 10.1111/cdoe.13019 (PMC11892544; doi:10.1111/cdoe.13019)
Supplement: Supplementary file 4 — Appendix S4. [file CDOE-53-180-s002.docx]

Appendix 4. Baseline and 2.5-year follow-up questions on motivational and volitional factors influencing toothbrushing

Table 1. Baseline questions on motivational and volitional factors influencing toothbrushing (self-efficacy, attitude, intention and coping and action planning) for randomised pupils (n=4680)

|  | **Intervention**  **(n=2262)** | **Control**  **(n=2418)** | **Overall**  **(n=4680)** |
| --- | --- | --- | --- |
| **Task self-efficacy, mean (SD)** | 3.4 (0.6) | 3.4 (0.6) | 3.4 (0.6) |
| **Attitudes, mean (SD)** | 3.2 (0.4) | 3.2 (0.4) | 3.2 (0.4) |
| **Coping planning, mean (SD)** | 2.7 (0.7) | 2.7 (0.7) | 2.7 (0.7) |
| **Action planning, mean (SD)** | 3.3 (0.6) | 3.3 (0.6) | 3.3 (0.6) |
| **Intention (How often do you want to brush your teeth?), n (%)** |  |  |  |
| >3x a day | 129 (5.7) | 138 (5.7) | 267 (5.7) |
| 3x a day | 443 (19.6) | 470 (19.4) | 913 (19.5) |
| Twice a day | 1488 (65.8) | 1591 (65.8) | 3079 (65.8) |
| Once a day | 132 (5.8) | 148 (6.1) | 280 (6.0) |
| <Once a day | 16 (0.7) | 20 (0.8) | 36 (0.8) |
| Never | 19 (0.8) | 16 (0.7) | 35 (0.7) |
| Missing | 35 (1.5) | 35 (1.4) | 70 (1.5) |

Table 2. Questions related to toothbrushing self-efficacy, attitude, intention and coping and action planning for randomised pupils at 2.5 years for pupils who completed this questionnaire (n=2643)

|  | **Intervention**  **(n=1287)** | **Control**  **(n=1356)** | **Overall**  **(n=2643)** |
| --- | --- | --- | --- |
| **Task self-efficacy, mean (SD)** | 3.5 (0.6) | 3.4 (0.6) | 3.4 (0.6) |
| **Attitudes, mean (SD)** | 3.3 (0.4) | 3.3 (0.4) | 3.3 (0.4) |
| **Coping planning, mean (SD)** | 2.7 (0.7) | 2.7 (0.7) | 2.7 (0.7) |
| **Action planning, mean (SD)** | 3.4 (0.6) | 3.4 (0.6) | 3.4 (0.6) |
| **Intention (How often do you want to brush your teeth?), n (%)** |  |  |  |
| >3x a day | 32 (2.5) | 28 (2.1) | 60 (2.3) |
| 3x a day | 217 (16.9) | 205 (15.1) | 422 (16.0) |
| Twice a day | 954 (74.1) | 1028 (75.8) | 1982 (75.0) |
| Once a day | 68 (5.3) | 78 (5.8) | 146 (5.5) |
| <Once a day | 6 (0.5) | 6 (0.4) | 12 (0.5) |
| Never | 5 (0.4) | 4 (0.3) | 9 (0.3) |
| Missing | 5 (0.4) | 7 (0.5) | 12 (0.5) |
